# Nonlinear properties of medial entorhinal cortex neurons reveal frequency selectivity during multi-sinusoidal stimulation

**DOI:** 10.3389/fncel.2014.00239

**Published:** 2014-08-19

**Authors:** Christophe Magnani, Michael N. Economo, John A. White, Lee E. Moore

**Affiliations:** ^1^CNRS UMR 8257, Université Paris DescartesParis, France; ^2^Department of Bioengineering, Brain Institute, University of UtahSalt Lake City, UT, USA

**Keywords:** entorhinal cortex, stellate neurons, grid cells, quadratic sinusoidal analysis, frequency domain, nonlinear oscillations, resonance

## Abstract

The neurons in layer II of the medial entorhinal cortex are part of the grid cell network involved in the representation of space. Many of these neurons are likely to be stellate cells with specific oscillatory and firing properties important for their function. A fundamental understanding of the nonlinear basis of these oscillatory properties is critical for the development of theories of grid cell firing. In order to evaluate the behavior of stellate neurons, measurements of their quadratic responses were used to estimate a second order Volterra kernel. This paper uses an operator theory, termed quadratic sinusoidal analysis (QSA), which quantitatively determines that the quadratic response accounts for a major part of the nonlinearity observed at membrane potential levels characteristic of normal synaptic events. Practically, neurons were probed with multi-sinusoidal stimulations to determine a Hermitian operator that captures the quadratic function in the frequency domain. We have shown that the frequency content of the stimulation plays an important role in the characteristics of the nonlinear response, which can distort the linear response as well. Stimulations with enhanced low frequency amplitudes evoked a different nonlinear response than broadband profiles. The nonlinear analysis was also applied to spike frequencies and it was shown that the nonlinear response of subthreshold membrane potential at resonance frequencies near the threshold is similar to the nonlinear response of spike trains.

## 1. Introduction

The stellate cells in layer II of the medial entorhinal cortex have long been noted for their oscillatory character (Erchova et al., 2004) consisting of membrane potential oscillations (MPOs) and resonance properties (Engel et al., 2008; Giocomo and Hasselmo, 2008; Pastoll et al., 2012; Shay et al., 2012). More recently, it has been suggested that these cells participate in the grid-like firing fields with regard to an animals position in space. In intact animals, grid cells have increased activity at particular locations representing a hexagonal grid. It is unclear how stellate cells along with pyramidal neurons participate in the grid cell networks (Burgalossi and Brecht, 2014; Ray et al., 2014). It has been proposed that nonlinear stochastic current fluctuations from ion channels, rather than an internal periodic oscillator, is responsible for this behavior (Erchova et al., 2004; Dodson et al., 2011). These neurons project to the hippocampus and are implicated in the activity of place cells that encode a single location. Thus, it is of some interest to understand in detail the nonlinear properties of stellate cells and how these are utilized in neural networks that compute spatial position.

Quantitative information about the linear and quadratic behaviors can be obtained from a current clamp of the soma, which provides a basis to understand the just threshold behavior of these neurons in a particular neural network. We have used multi-sinusoidal stimulations to elicit stellate neuronal quadratic responses in the frequency domain, which are then encoded into a matrix representing a Hermitian operator. The underlying theory, termed quadratic sinusoidal analysis (QSA), was published by Magnani and Moore (2011) for voltage clamped neurons and in this paper, is applied to current clamp. The QSA not only quantitatively characterizes experimental data from stellate cells, but it also provides an evaluation of the corresponding conductance based models.

The method of frequency probing used here is based on a practical measurement technique, namely harmonic probing on Volterra kernels (Victor and Shapley, 1980; Boyd et al., 1983). Multi-sinusoidal stimulations enable nonlinear measurements of the neuronal response at harmonics 2 *f_i_* and interactive frequencies |*f_i_* ± *f_j_*|. These measurements constitute the coefficients of the QSA matrix, which represents an operator as an algebraic object similar to a Volterra kernel rather than just an array of numbers. It provides a precise signature of the nonlinear voltage dependent conductances and their particular representation on the dendritic and somatic membranes.

Importantly, these studies show in detail how quadratic properties of neurons are dependent on the frequency content of the stimulation and demonstrate that the nonlinear behavior accurately describes membrane potential responses at the level of normal synaptic activity (1–5 mV).

## 2. Materials and methods

### 2.1. Tissue preparation

All experimental protocols were approved by the Boston University and University of Utah Institutional Animal Care and Use Committees. Horizontal sections of entorhinal cortex were prepared from 14- to 28 d-old LongEvans rats. All chemicals were obtained from Sigma (St. Louis, MO) unless otherwise noted. After anesthetization with isoflurane and euthanasia, brains were removed and immersed in 0°C artificial CSF (ACSF) consisting of the following (in mM): 125 NaCl, 25 NaHCO_3_, 25 D-glucose, 2 KCl, 2 CaCl_2_, 1.25 NaH_2_PO_4_, 1 MgCl_2_, and buffered to pH 7.4 with 95/5% O_2_/CO_2_. Horizontal slices were cut to a thickness of 400 μm (Vibratome 1000+; Vibratome, St. Louis, MO). Slices were incubated in a 32°C bubbled ACSF for 30 min before being cooled to room temperature (20°C). After the incubation period, slices were moved to the stage of an infrared, differential interference contrast-equipped microscope (Axioscope 2+; Zeiss, Oberkochen, Germany). All recordings were conducted between 32 and 34°C. Solutions and preparation were identical to those described in Fernandez and White (2008).

### 2.2. Electrophysiology

Electrodes were drawn on a horizontal puller (P97; Sutter Instruments, Novato, CA) and filled with an intracellular solution consisting of the following (in mM): 120 K-gluconate, 20 KCl, 10 HEPES, 7 diTrisPhCr, 4 Na_2_ATP, 2 MgCl_2_, 0.3 Tris-GTP, and 0.2 EGTA, buffered to pH 7.3 with KOH. Final electrode resistances were between 3 and 4 MΩ, with a range of measured access values between 4 and 12 MΩ. All recordings were taken from the medial entorhinal cortex (MEC). Stellate cells were identified as neurons within layer II of the MEC exhibiting a large sag profile in response to hyperpolarizing current and having a peak between 2 and 7 Hz in their subthreshold impedance spectrum. Electrophysiological recordings were performed with a current-clamp amplifier MultiClamp 700A; Molecular Devices, Union City, CA), and data were acquired using custom software developed in MATLAB 2014 (MathWorks, Natick, MA) using the data acquisition toolbox. All additional details of the electrophysiological measurements are given in Fernandez et al. (2013).

### 2.3. Theoretical simulations

The simulations were done by implementing Hodgkin-Huxley type conductance based models in MATHEMATICA 8 and 9 (Wolfram Research, Champaign, IL, USA). The parameter estimation methods for all models is identical to that used for experiments on prepositus hypoglossi neurons in Idoux et al. (2008). The model structure consists of a soma and eight dendritic compartments with uniform distributions for three voltage dependant ionic conductances : persistent sodium *g*_NaP_, potassium *g*_K_ and hyperpolarization activated conductance *g*_H_. In particular, since action potentials are not being simulated, the total sodium conductance is treated as one non-inactivating conductance *g*_NaP_. The gating variables are considered without power functions. Thus, the nonlinear behavior is essentially due to rate constants rather than power functions. Statistics were done with the PairedZTest of the Hypothesis Testing package of MATHEMATICA.

### 2.4. QSA theory

Linear systems are completely characterized by the linear superposition principle, which means that the response to a linear superposition of sine waves is a linear superposition of sine waves with the same frequencies but different amplitudes and phases. In contrast, the response of a nonlinear system can have frequencies not present in the stimulation. More precisely, if a multi-sinusoidal stimulation has frequencies *f_i_* and *f_j_* then the linear response will have frequencies *f_i_* and *f_j_* whereas the quadratic response will have additional harmonics 2 *f_i_*, 2 *f_j_* and interactive frequencies |*f_i_* ± *f_j_*|. There also exist higher order interactions, such as *f_i_* + *f_j_* + *f_k_*, however the neurons studied in this paper mainly manifest quadratic nonlinearities. In particular, multi-sinusoidal stimulations require small amplitudes such that only linear and quadratic responses are significant, although the amplitudes must be large enough to overcome the background noise.

A major obstruction to experimental measurements of nonlinear responses is due to frequency overlaps. This means that two (or more) input frequencies can generate the same output frequency. For example, the input frequencies 1, 2, 3, 4 (in Hz) generate ambiguous output frequencies such as 3 − 1 = 4 − 2 (in Hz). A solution consists of choosing carefully the input frequencies without overlap up to the second order, higher orders being assumed negligible. An algorithm was written by Magnani and Moore (2011) to generate nonoverlapping frequencies.

The Fourier transform applied to a multi-sinusoidal current stimulation *I*(*t*) generates Fourier coefficients at input frequencies *f_i_*. The Fourier transform applied to the membrane potential *V*(*t*) generates Fourier coefficients at input frequencies *f_i_* as well as at harmonics 2 *f_i_* and interactive frequencies |*f_i_* ± *f_j_*|. In linear analysis, the Fourier coefficients of *I*(*t*) and *V*(*t*) can be used to compute the linear transfer function (impedance) *Z*[*f_k_*] = *V*[*f_k_*]/*I*[*f_k_*]. Following Magnani and Moore (2011), we compute the quadratic transfer function

(1) $$B_{ij}=\gamma_{ij}\frac{V[f_{i}+f_{j}]}{I[f_{i}]I[f_{j}]}$$

where γ_*ii*_ = 1 and γ_*ij*_ = 1/2 for *i* ≠ *j*. The combinations *f_i_* + *f_j_* include all sums and differences between |*f_i_*| and |*f_j_*| when considering both positive and negative frequencies. The frequencies *f_k_* are indexed over the ordered set of integers Γ = {−*N*, …, −1, +1, …, +*N*}.

Importantly, Magnani and Moore (2011) have shown that the complex matrix *B* can be turned into a Hermitian matrix *Q*, termed the QSA matrix, by row flipping of the coefficients *Q*_*i*,*j*_ = *B*_−*i*,*j*_. Although a Hermitian matrix has complex coefficients, its eigenvalues are real numbers, which are much easier to interpret physically as amplitudes in mV/nA^2^. In this way, the quadratic part of the membrane potential can be expressed as an algebraic formula

$$V_{2}(t)=I_{t}^{*}QI_{t}$$

where *I_t_* is a time dependent vector encoding the multi-sinusoidal stimulation.

The quadratic response can be reduced to a sum of squares through eigenanalysis of *Q*

$$V_{2}(t)=\sum_{i\in \Gamma }d_{i}|w_{i}{|}^{2}$$

where *d_i_* are eigenvalues (mV/nA^2^) and |*w_i_*|^2^ are stimulation components (nA^2^) obtained from a linear transformation (Magnani and Moore, 2011). In this way, the quadratic neuronal function can be interpreted as a set of quadratic filters where eigenvalues are amplitudes. When an eigenvalue is dominant and others are small, the quadratic neuronal function is approximately a simple square.

Bode plots are useful to represent amplitude with respect to frequency. The QSA matrix can be represented in this way by summing the amplitude components of each column. However, unlike eigenanalysis, this induces information loss. The *R* summation function (mV/nA^2^) is defined by

(2) $$R_{j}=\sum_{i\in \Gamma }|Q_{ij}|$$

Alternatively, a modified function *R*' with units homogenous to a linear transfer function (mV / nA) is defined by

(3) $${R^{\prime }}_{j}=\sum_{i\in \Gamma }|Q_{ij}|\cdot |I_{i}|$$

Intuitively, the *R* and *R*' functions evaluate the sum of the amplitudes at all frequencies for which they interact.

**Figure 1:**
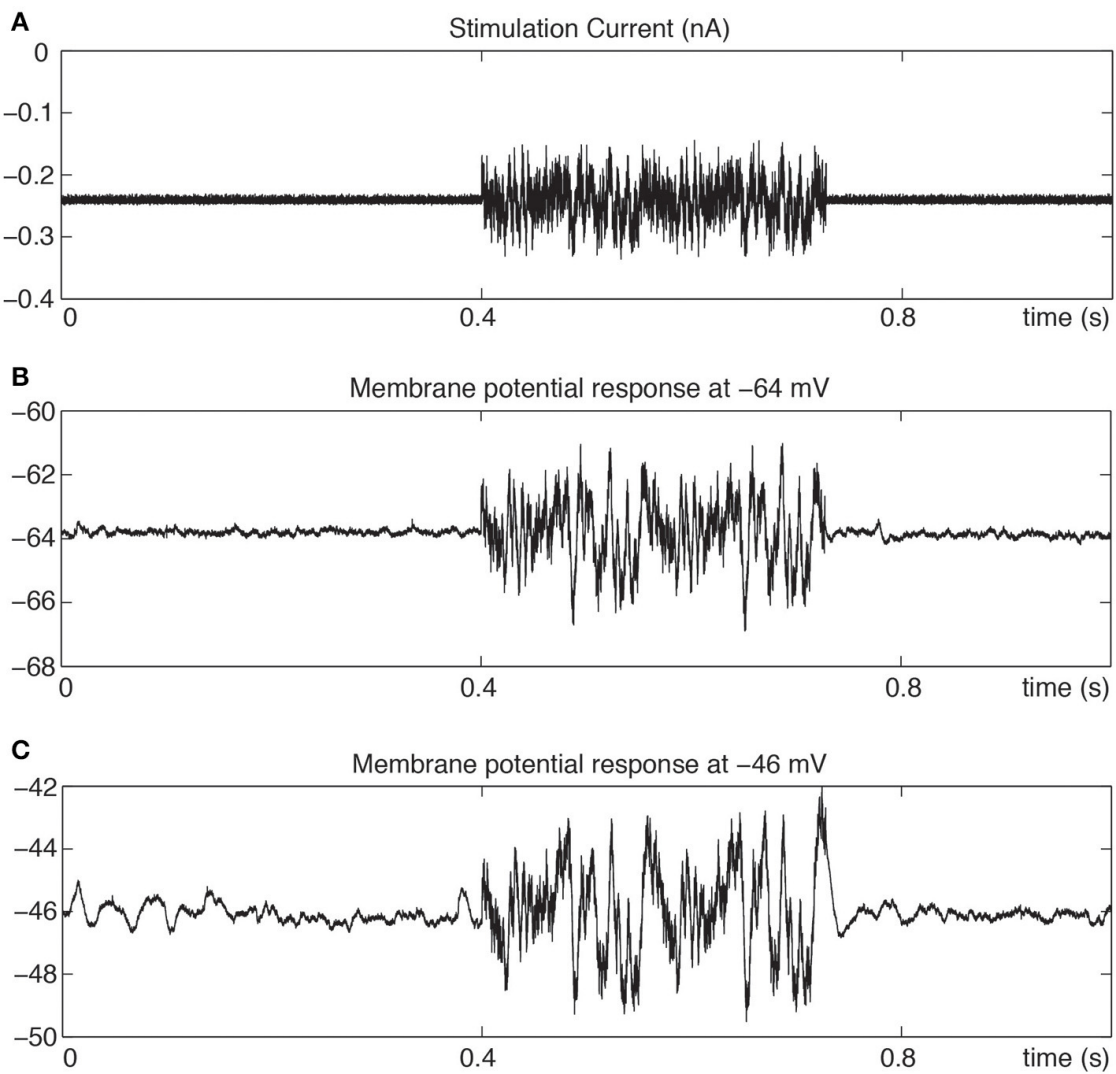
**Membrane potential response to multi-sinusoidal current stimulation with holding current adjusted to give −64 and −46 mV average membrane potentials. (A)** Stimulation current with range of frequencies from 0.2 Hz to 2 kHz. **(B)** Membrane potential response at −64 mV. **(C)** Membrane potential response at −46 mV with spontaneous oscillations preceding the stimulation.

Figure 1 illustrates the membrane potential response of a stellate neuron to a multi-sinusoidal current stimulation at two membrane potential levels. Membrane potential oscillations (MPOs) are clearly observed before and after the stimulation at the depolarized level. Also, the response shows larger oscillations during the depolarization. This neuron has a strong resonance in the same frequency range as the MPO's (Schreiber et al., 2004; Engel et al., 2008; Yoshida et al., 2011).

**Figure 2:**
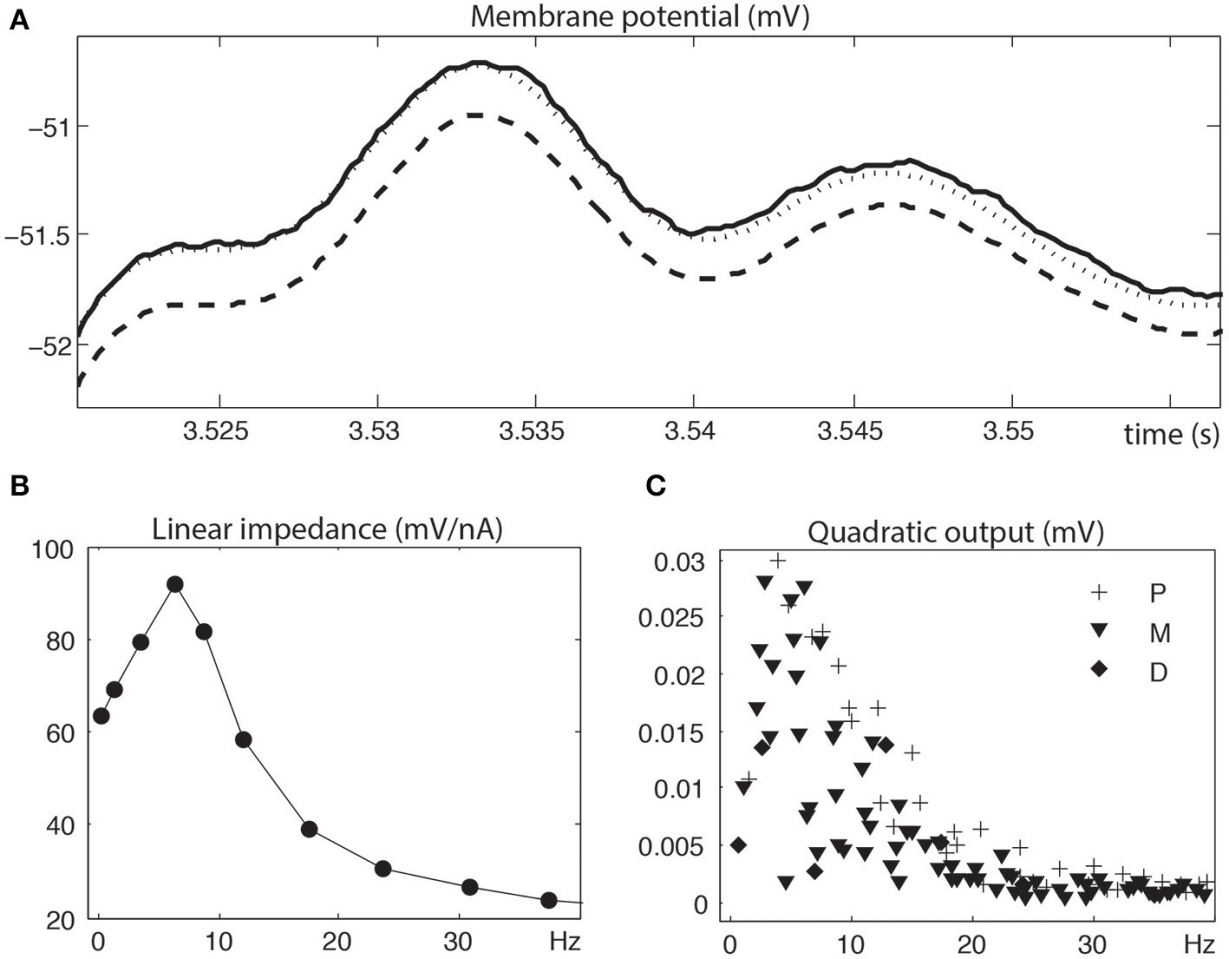
**Linear and quadratic analyses of a depolarized stellate neuron. (A)** Zoomed measured membrane potential (solid), linear reconstruction (dashed) and linear + quadratic reconstruction (dotted). **(B)** Linear impedance at input frequencies (circle symbol at *f_i_*). **(C)** Quadratic output at harmonics (diamond symbol at 2 *f_i_*), interactive sums (plus symbol at *f_i_* + *f_j_*) and interactive differences (inverted triangle symbol at |*f_i_* − *f_j_*|). The linear and quadratic analyses were truncated at 40 Hz in order to better illustrate the larger responses at the lower frequencies. Stimulation frequencies are 0.3, 1.3, 3.5, 6.4, 8.7, 12.1, 17.5, 23.7, 30.8, 37.4, 42, 51.3, 60.2, 74.9, 85.9 (in Hz).

Figure 2 illustrates the linear and quadratic analyses of a depolarized stellate neuron. Figure 2A compares the measured membrane potential in the time domain to the linear reconstruction and linear + quadratic reconstruction. Clearly, for these signal levels, linear analysis is insufficient and quadratic analysis is required to capture the nonlinear neuronal behavior. Figures 2B,C show the linear impedance (mV/nA) and the quadratic output (mV) in the frequency domain. The Fourier components of the linear impedance occur at the stimulation frequencies, whereas those of the quadratic output occur at second order harmonics and interactive frequencies. Clearly, the quadratic output shows a strong overlap with the linear resonance frequencies.

The QSA theory applied to neurons does not rely on any assumption about their morphology or ion channel distribution. In particular, the complexity of Hodgkin-Huxley based multi-compartmental models with their computational overhead is avoided. QSA analyses were done with MATLAB on both model simulations and experimental data, allowing precise comparisons of the quadratic responses.

## 3. Results

### 3.1. Effect of membrane potential

**Figure 3:**
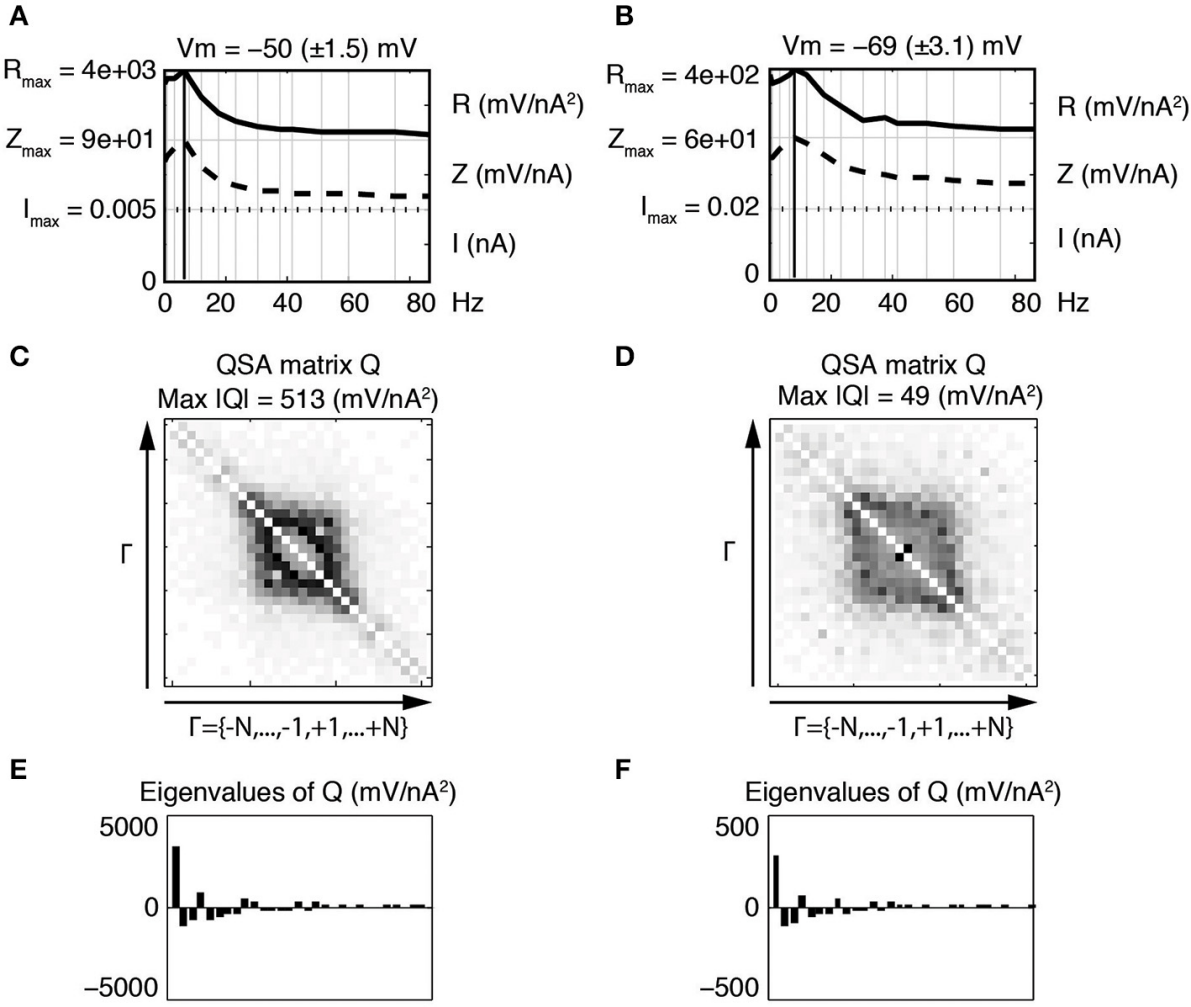
**Effect of the membrane potential level on linear and quadratic responses of a stellate neuron at −50 mV (left column) and −69 mV (right column)**. The standard deviation (STD) is indicated in parenthesis. The frequencies are indexed as *f_k_* where *k* ∈ Γ = {−*N*, …, −1, +1, …, +*N*} and *N* = 15 denotes the number of stimulation frequencies. **(A,B)** Juxtaposed plots with respect to the input frequencies, namely the amplitudes of current stimulation *I*, linear impedance *Z*, and *R* function. A vertical bold line indicates the resonance frequency, which is *f*_4_ = 6.4 Hz for **(A)** and *f*_5_ = 8.7 Hz for **(B)**. The amplitude for each plot ranges from zero to the indicated maximum. Each maximum coincides with zero for the next juxtaposed plot. **(C,D)** Amplitudes of the coefficients of the QSA matrix *Q*, where the darkest rectangle represents the maximum value given by Max |*Q*| in the subtitle. Each matrix is indexed by Γ ordered from negative to positive numbers in the direction of the arrows. For example, column *f*_6_ and row *f*_−3_ represents the frequency interaction *f*_6_ − *f*_3_ = 12.1 − 3.5 = 8.6 Hz. **(E,F)** Eigenvalues of the QSA matrix ordered by decreasing amplitudes. Stimulation frequencies are 0.3, 1.3, 3.5, 6.4, 8.7, 12.1, 17.5, 23.7, 30.8, 37.4, 42, 51.3, 60.2, 74.9, 85.9 (in Hz).

Figure 3 illustrates the linear and quadratic responses of a stellate neuron for two membrane potentials −50 and −69 mV (left and right columns respectively). The stimulation frequencies were identical to those of Figure 2 and are indexed as *f_k_* where *k* ∈ Γ = {−15, …, −1, +1, …, +15}. The upper panels Figures 3A,B represent a juxtaposition of the amplitudes for the stimulations *I*, linear impedances *Z*, and *R* functions with respect to the input frequencies. The *R* functions are plotted as Bode plots in the same way as the impedances although the ordinate units are different. The maximum of each *R* function is close to the impedance resonance frequency. Statistics were calculated for a group of six stellate neurons. The maximum amplitude of the QSA matrix increased from 275 to 715 mV/nA^2^ (*p* = 0.0004) for a membrane potential change of +7 mV in the range −65 to −48 mV.

As explained in previous publications (Magnani and Moore, 2011; Magnani et al., 2013), the QSA matrix provides a complete description of the quadratic response as ratios between output and input coefficients (Equation 1). In contrast, the coded points in Figure 2C show the quadratic measurements of the output without showing the frequency interactions (*f_i_*, *f_j_*). The QSA matrix was constructed from the Fourier coefficients of the data, then decomposed by eigenanalysis to compute its eigenvalues. Each cell of the QSA matrix represents the amplitude of the voltage response at two interactive frequencies divided by the amplitude of the current at these two frequencies (Equation 1). The matrix plots have many symmetries, which reflect particular algebraic properties of the underlying neural operator. The matrix plot is indexed by the set Γ ordered from negative to positive numbers in the direction of the arrows, that is to say *f*_−15_, …, *f*_−1_, *f*_+1_, …, *f*_+15_. Each cell at abscissa *f_i_* and ordinate *f_j_* encodes the ratio between the membrane potential at *f_i_* + *f_j_* and the current at *f_i_* and *f_j_*. In particular, the white diagonal encodes interactions *f_i_* + *f*_−*i*_ = 0, thus the DC is set to zero. The other diagonal *f_i_* + *f_i_* encodes the harmonics 2 *f_i_*. In Figures 3C,D, the QSA amplitudes appear concentrated at lower frequencies near the center of the matrix, which is consistent with the maximum of the *R* function.

The impedance and resonance frequency are much less dependent on the membrane potential than the QSA matrix and *R* function. This suggests that the quadratic neuronal function especially encodes nonlinear voltage dependent ionic conductances. The effect of the membrane potential on stellate neurons is pronounced for all nonlinearities, namely the amplitudes of the QSA coefficients, the eigenvalues and the *R* functions.

In this and all subsequent figures, the *R* function (mV/nA^2^) is juxtaposed on the linear impedance (mV/nA), which in turn is juxtaposed on the stimulation amplitude Fourier spectrum (nA). Although the *R* function is a non reversible reduction of the QSA matrix, it provides a practical way to compare the linear and quadratic behaviors at input frequencies. It can be observed, in Figures 3A,B, that the *R* function has a resonance frequency range comparable (but not identical) to the linear case.

At −50 mV (Figure 3, left column), the QSA matrix gives more detail on frequency interactions showing enhanced amplitudes in the centered square delimited by |*f*_± 4_| = 6.4 Hz, namely those that involve the resonance frequencies. At the more hyperpolarized membrane potential −69 mV (Figure 3, right column), the QSA matrix shows lower amplitudes. However, there is a peak for the harmonics of the lowest input frequency 2 *f*_1_ = 0.6 Hz. Moreover, the quadratic response is enhanced around the centered square delimited by the slightly higher resonance frequency *f*_5_ = 8.7 Hz. Thus, the shift in frequency of the nonlinear responses with membrane potential level is similar to the voltage dependence of the linear resonance frequencies (Shay et al., 2012).

**Figure 4:**
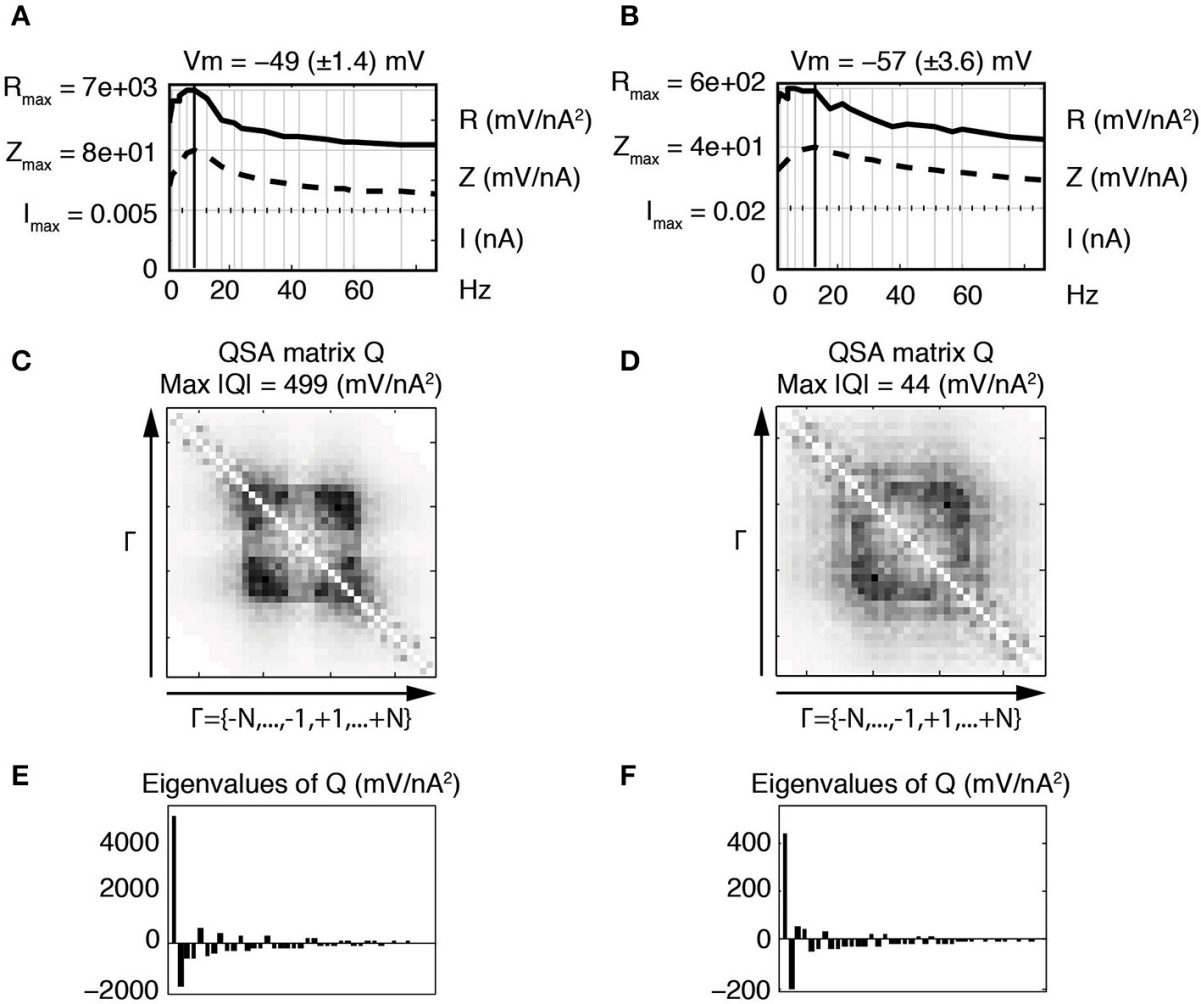
**Effect of the membrane potential level on linear and quadratic responses of stellate neuronal model simulations at −49 mV (left column) and −57 mV (right column). (A,B)** Juxtaposed plots with respect to the input frequencies, namely the amplitudes of current stimulation *I*, linear impedance *Z*, and *R* function. The resonance frequencies are respectively *f*_8_ = 8.7 Hz **(A)** and *f*_9_ = 12.1 Hz **(B)**. **(C,D)** Amplitudes of the coefficients of the QSA matrix *Q* with the maximum value given by Max |*Q*|. **(E,F)** Eigenvalues of the QSA matrix. The model parameter values (in units based on mV and nA) are as follows : membrane capacitance *C*_soma_ = 0.0000542, maximal conductances *g*_leak_ = 0.0005, *g*_K_ = 0.023, *g*_NaP_ = 0.0024 and *g*_H_ = 0.014; the reversal potentials *V*_leak_ = −55, *V*_K_ = −87, *V*_NaP_ = 77 and *V*_H_ = −43. The functions α*_n_*, β*_n_*, α*_m_*, β*_m_*, α*_h_*, β*_h_* depend on the variable *V* and their description is given by Idoux et al. (2008) : *v_n_* = −35, *s_n_* = 0.045, *t_n_* = 0.7543, *v_m_* = −38, *s_m_* = 0.06, *t_m_* = 0.000150, *v_h_* = −51.65, *s_h_* = −0.06 and *t_h_* = 0.05. Finally, the electronic length is *e*_length_ = 0.50 and the ratio of the dendritic area to the soma area is *A*_ratio_ = 4.5. Stimulation frequencies are 0.3, 0.4, 1.3, 1.5, 3.5, 4.0, 6.4, 8.7, 12.1, 17.5, 21.6, 23.7, 30.8, 37.4, 42.0, 51.3, 56.9, 60.2, 74.9, 85.9 Hz (in Hz).

The eigenanalysis of the QSA matrix reveals that the nonlinear function is concentrated in a single dominant eigenvalue. This suggests that the neuronal processing consists of a single nonlinear-linear unit as opposed to a parallel combination of several units (see Magnani and Moore, 2011). Dominant eigenvalues are frequently observed, however there are generally multiple significant eigenvalues. Figure 4 illustrates that a simplified model of stellate neurons is able to capture the nonlinear behavior described by experiments of Figure 3.

### 3.2. Linear distortions

Previous experiments by Haas and White (2002) suggested that the Fourier responses of stellate neurons to multiple frequencies were dependent on the frequency content of the stimulation, and furthermore these responses were different from that obtained when using single sine waves. We have explored this issue in more detail by comparing the responses of stellate neurons to stimulations containing many frequencies vs. QSA stimulations containing nonoverlapping frequencies. The presumed linear responses in the mV range to many frequencies show marked distortions compared to almost no distortions using nonoverlapping frequencies. The term, distortions, is used here to describe an irregular function with larger variations than generally seen in our linear impedance functions.

**Figure 5:**
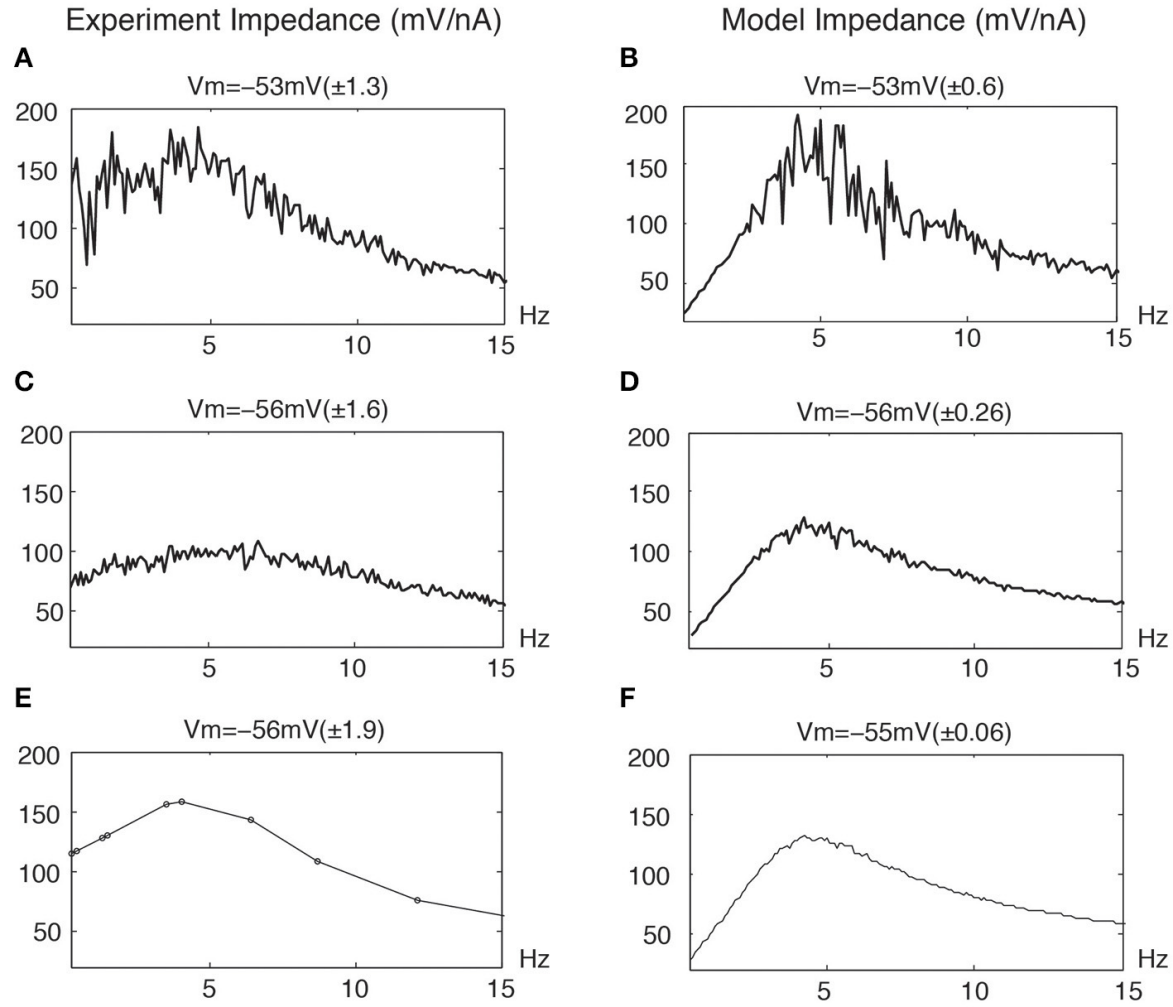
**Distortion of linear impedance (mV/nA) by the stimulation profiles for experimental data and model simulations**. The average membrane potential *V*_m_ is given along with STD values of membrane potential. The standard deviations are given in parenthesis. **(A,C)** Experimental data : the same overlapping frequencies in 0.3 − 1000 Hz with 0.1 Hz intervals. **(E)** Experimental data : the nonoverlapping frequencies are the same as Figure 4. **(B,D,F)** Model simulations : the same overlapping frequencies in 0.3 − 500 Hz with 0.1 Hz intervals. The model parameters are given in **Figure 7**.

Figures 5A,C,E (left column) illustrates distortions in the frequency responses from stellate neurons due to nonlinear responses at overlapping frequencies. The upper trace (A) shows a marked distortion at −53 mV of linear responses stimulated with a very large number of frequencies near threshold. The middle trace (C) shows a significant reduction in the distortion of the linear response for a more hyperpolarized membrane potential with the same number of overlapping frequencies. Finally, the bottom trace (E) shows an almost undistorted linear response using nonoverlapping frequencies (up to the second order) despite a greater membrane potential STD.

Clearly, stimulations with overlapping frequencies generate interactive frequencies that overlap the input frequencies and thereby cause a distortion in the linear response. These effects naturally increase with the number and amplitudes of the particular frequencies. Similarly, Figures 5B,D,F (right column) illustrates similar distortions from a neuronal model. The upper panel (B), near threshold, shows that the linear response is significantly distorted due to the presence of nonlinear interactive frequencies for potential excursions in the mV range. The middle panel (D) shows less but distinct distortions at a lower STD and a more hyperpolarized membrane potential, which is consistent with the experimental results (left middle panel). The bottom panel (F) shows an almost undistorted linear response with the same overlapping frequencies for a still lower STD membrane potential.

Generally, if the output amplitude STD using many overlapping frequencies is less than 0.5 mV, then the linear responses have minimal distortion. Undistorted linear responses can be obtained with fewer nonoverlapping frequencies for much higher STD membrane potential responses, typically up to 4 mV. Thus, a larger number of overlapping frequencies evoking responses in the mV range induce significantly more nonlinear effects than fewer nonoverlapping frequencies. Nonstationary signals, such as the chirp or the zap, are not optimal for linear analysis since their Fourier components distribute over all frequencies and dramatically generate frequency overlaps. Nevertheless, linear analysis is still possible for small stimulation amplitudes (Erchova et al., 2004; Schreiber et al., 2004).

### 3.3. Low pass stimulation filtering

The above results suggest that the effect of the frequency content of the stimulation on the true nonlinear response should be distinguished from the linear distortions. Experiments were done with a low pass filtered stimulation using nonoverlapping frequencies in which the high frequency amplitudes are reduced. Nonoverlapping frequencies assure that the linear responses are not distorted in order to accurately measure the nonlinear behavior. Since stellate neurons have resonance frequencies around 10 Hz or lower, it would not be surprising that these frequencies compared to high frequencies would have the largest amplitude responses and thus can easily evoke the nonlinear behavior.

The nonlinear responses induced by differently filtered Gaussian white noise inputs are likely to be stimulation dependent. This not only leads to nonlinear frequency interactions dependent on the frequency content of the stimulation, but linear analysis can also be altered since Fourier components at individual input frequencies would be contaminated by nonlinearities. Surprisingly these effects can occur at relatively small amplitudes of membrane potential responses, namely in the mV range. Thus, empirically, responses of neurons at their stimulating frequencies can be dependent on the frequency content of the stimulation, although a true linear response is not.

**Figure 6:**
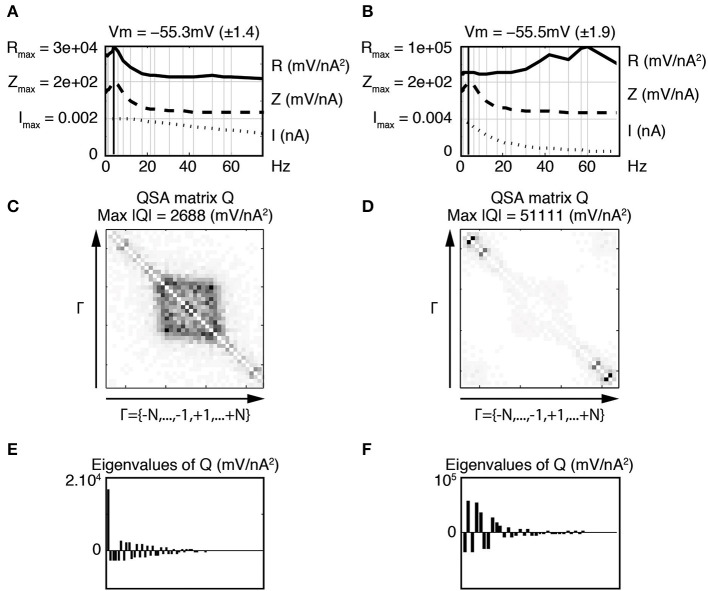
**Effect of stimulation profile on stellate neurons**. Left column **(A,C,E)** Weakly low pass filtered stimulation *I* in dotted lines. Right column **(B,D,F)** Strongly low pass filtered stimulation *I* in dotted lines. The resonance of the impedance (*Z*) is *f*_6_ = 4.0 Hz for both stimulations **(A,B)**. The attenuation of high frequencies in the stimulation of the second column induces enhanced quadratic responses as indicated by the *R* functions in the top row **(A,B)** as well as the QSA matrix **(C,D)** and eigenvalues **(E,F)**. The stimulation frequencies are the same as in Figure 4.

Figure 6 shows that the nonlinear responses of a stellate neuron to various low pass filtered stimulations are remarkably different despite identical linear behaviors. The cutoff frequencies are not the same between left and right columns, showing that the increase of high frequency interactions are related to high frequency filtering. Statistics were calculated for a group of three stellate neurons comparing the effect of the two low pass stimulation filters. The maximum amplitude of the QSA matrix increased from 1229 to 4204 mV/nA^2^ (*p* = 0.019) for a membrane potential change of −60 to −55 mV.

The coefficients of the QSA matrix are defined as the ratios between an interactive output and the product of the corresponding inputs for each pair of input frequencies. Thus, although the amplitudes of the high frequency responses decrease when the stimulation is filtered, the above ratio tends to increase. Therefore, the quadratic function appears sensitive to the frequency content of the stimulation even for membrane potential responses in the mV range.

**Figure 7:**
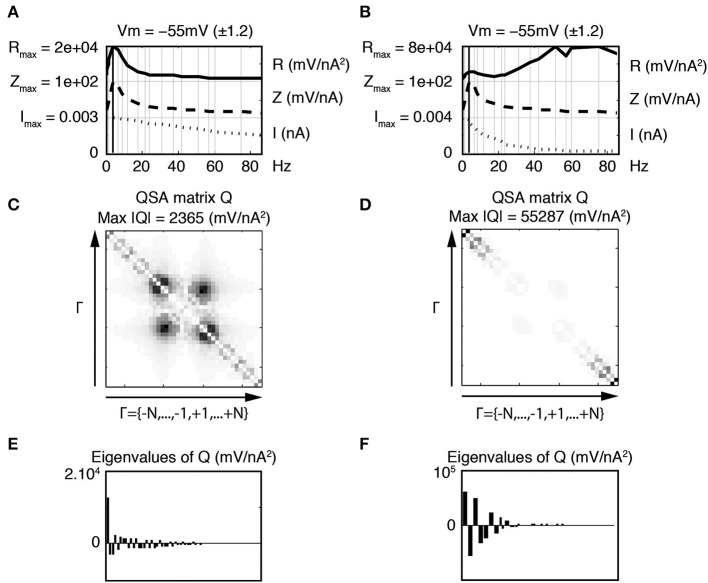
**Effect of stimulation profile on stellate neuronal model simulations**. Left column **(A,C,E)** Weakly low pass filtered stimulation *I* in dotted lines. Right column **(B,D,F)** Strongly low pass filtered stimulation *I* in dotted lines. The resonance of the impedance (*Z*) is *f*_6_ = 4.0 Hz for both stimulations **(A,B)**. The attenuation of high frequencies in the stimulation of the second column induces enhanced quadratic responses as indicated by the *R* functions in the top row **(A,B)** as well as the QSA matrix **(C,D)** and eigenvalues **(E,F)**. The stimulation frequencies are the same as in Figure 4. The parameter values (in units based on mV and nA) are as follows : membrane capacitance *C*_soma_ = 0.0000542, maximal conductances *g*_leak_ = 0.0005, *g*_K_ = 0.0627, *g*_NaP_ = 0.0065 and *g*_H_ = 0.002926; the reversal potentials *V*_leak_ = −55, *V*_K_ = −87, *V*_NaP_ = 77 and *V*_H_ = −43. The functions α*_n_*, β*_n_*, α*_m_*, β*_m_*, α*_h_*, β*_h_* depend on the variable *V* and their description is similar to those published by Idoux et al. (2008) : *v_n_* = −38, *s_n_* = 0.035, *t_n_* = 0.75, *v_m_* = −38.76, *s_m_* = 0.046, *t_m_* = 0.000150, *v_h_* = −51.65, *s_h_* = −0.01 and *t_h_* = 0.5567. Finally, the electronic length is *e*_length_ = 0.50 and the ratio of the dendritic area to the soma area is *A*_ratio_ = 4.5.

Increases in the *R* functions at high frequencies are clearly observed, however the QSA matrix gives more detail about the interactive frequencies. The QSA matrix of the left column shows peak amplitudes for the harmonics of input frequencies near the linear resonance frequency, such as 2 *f*_6_ = 8 Hz. Moreover, there are significant interactive differences between high input frequencies such as *f*_19_ − *f*_18_ = 14.7 Hz. The QSA matrix of the right column shows greatly enhanced high frequency interactions in response to the high frequency filtered stimulation. Although the lower frequency interactions near the resonance are not apparent, their amplitudes are similar as can be seen from the *R* functions. The complexity of these responses is also reflected through eigenanalysis that turns a single dominant eigenvalue to multiple eigenvalues when the stimulation is filtered. Figure 7 illustrates similar results for model simulations. It will be shown below that the increases of the responses at interactions between high frequencies are quite sensitive to the relative amplitude ratio of the low vs. high frequency stimulation content.

The stimulation dependence of the nonlinear response is a good example of the dramatic difference between linear and nonlinear behaviors even at small signal amplitudes. Linear responses are independent of stimulation amplitude for all frequencies. However, we have shown that interactive differences between high frequencies are not proportional to the stimulation amplitude when sufficiently low pass filtered. The quadratic transfer function is defined as the ratio between an interactive output and the product of two inputs. When the enhanced interaction corresponds to a difference between high frequencies, the ratio tends to be maximized because the numerator reflects a significant nonlinear response and the denominator is small due to the low pass filtering.

### 3.4. Band pass stimulation filtering

The above results demonstrate that the frequency content of the stimulation plays an important role in the nature of the nonlinear response, but generally has minimal effects on the linear behavior if the membrane potential responses are less than 0.5 mV. In order to determine if nonlinear responses have limiting small signal responses that are independent of the content of the stimulation, model simulations were done by applying a Gaussian-like window to the stimulation in the frequency domain. In this way, the stimulation shows a peak amplitude at a specific frequency (like a resonance).

**Figure 8:**
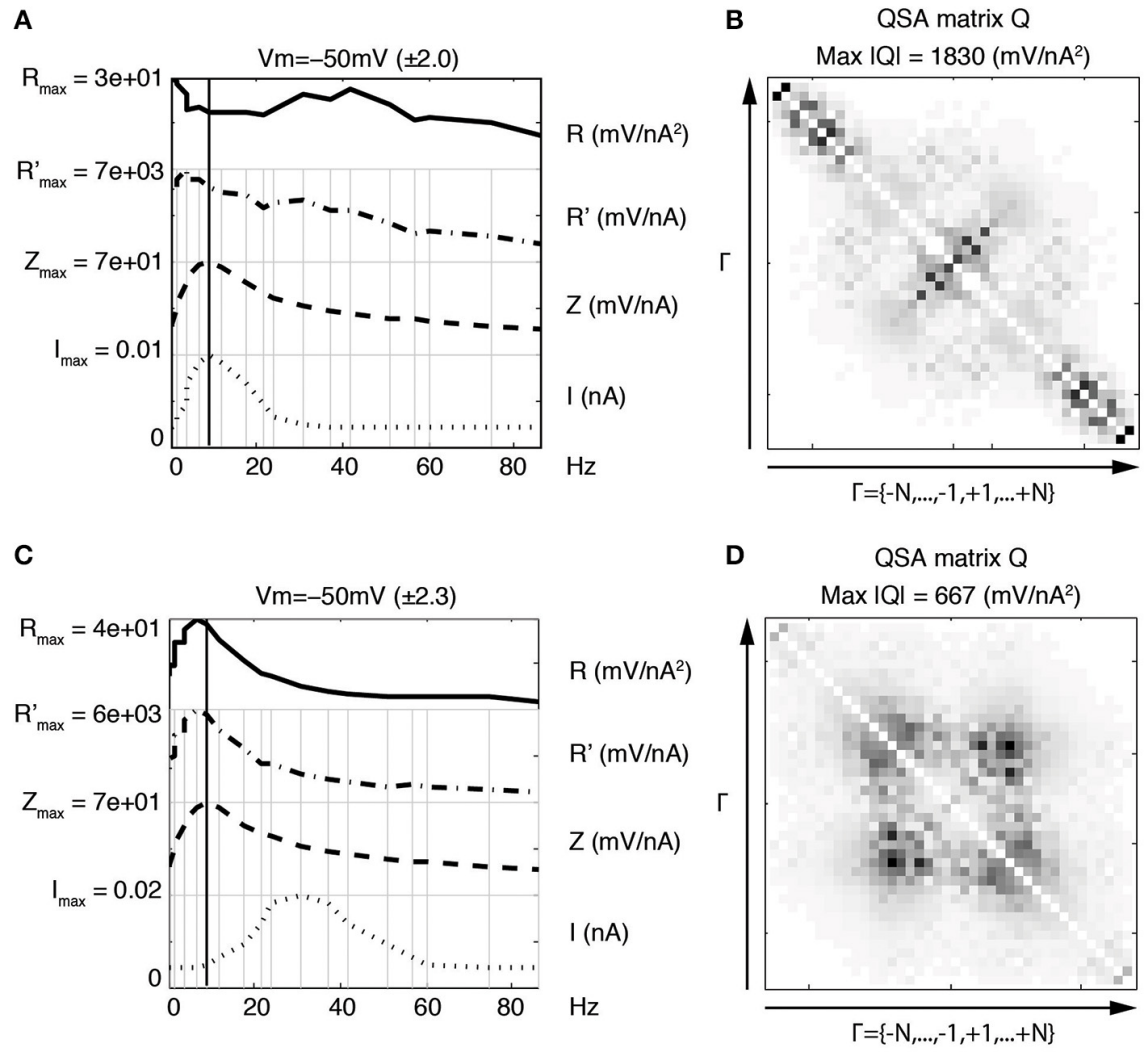
**Nonlinear effects of a Gaussian-like window applied to the stimulation Fourier components**. Upper row **(A,B)** Gaussian-like window applied to the stimulation Fourier components near the resonance *f*_8_ = 8.7 Hz. This induces enhanced frequency interactions between input frequencies near the resonance. Lower row **(C,D)** Gaussian-like window applied to the stimulation Fourier components three fold above the resonance *f*_8_ = 8.7 Hz. The QSA matrix appears much smaller than for a stimulation peak near the resonance.

Figure 8 illustrates that, in contrast to the linear impedance, if the stimulation peak is at a low frequency (panel A, near the resonance), then the maximum of the QSA matrix is much greater than for a stimulation peak at a higher frequency (lower row, beyond the resonance). Both stimulations evoke potential responses in the mV range and have identical linear impedances.

Discrete peaks can also be observed for the QSA matrix in Figures 8B,D. The panel B shows discrete peaks close to the harmonics of *f*_4_ = 1.5 Hz as well as high frequency interactions in the corners. The panel D also shows discrete peaks clustered around harmonic frequencies of *f*_7_ = 6.4 Hz and *f*_8_ = 8.7 Hz. In summary, Gaussian-like window applied near the resonance (panel A) evokes output frequencies before and after *f*_8_ = 8.7 Hz. Gaussian-like window applied after the resonance (panel C) leads to output frequencies that are near the resonance. Therefore, nonlinear responses involve a different frequency range compared to the linear resonance frequency.

**Figure 9:**
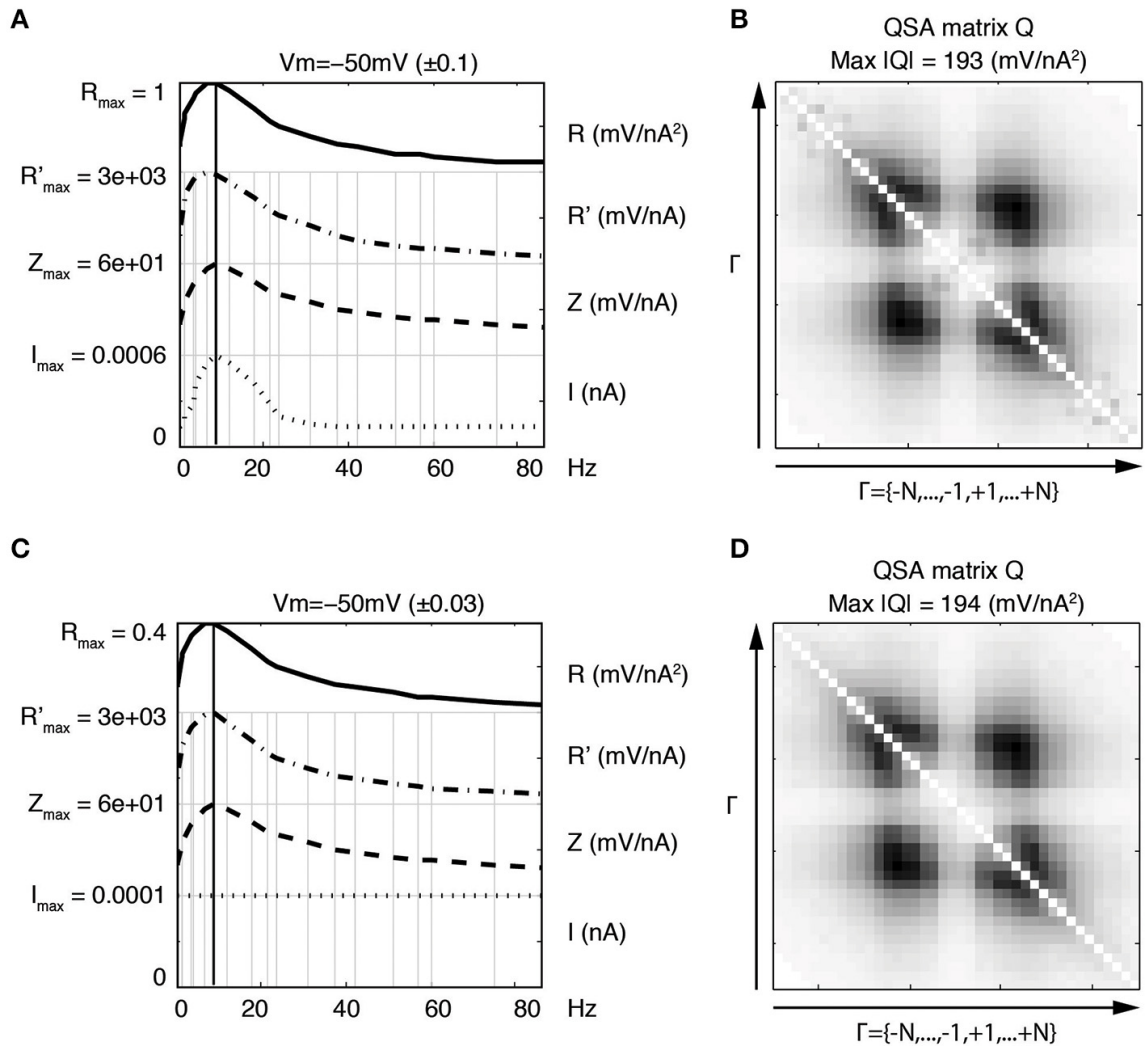
**Equivalence of nonlinear responses for different stimulation profiles at very small amplitudes**. Upper row **(A,B)** Gaussian-like window applied to the stimulation Fourier components. Lower row **(C,D)** constant amplitude stimulation profile. The linear and nonlinear responses are nearly the same, however the maximum of the *R* function is about two fold higher for the Gaussian-like stimulation.

Figure 9 illustrates that the quadratic response to very small stimulations is almost independent of the frequency content. In particular, both linear and quadratic functions are nearly identical for flat stimulation vs. low peak stimulation, as well as higher peak stimulation (not shown). Thus, the enhanced corners for low peak stimulation almost disappear if the membrane potential responses have STD values of 0.1 mV or less. In particular, the QSA matrix has a remarkable sensitivity to stimulations with low frequency content, especially near the resonance of the neuron. Indeed, Figure 9A shows a larger *R* function for such nonuniform stimulation profile.

From a mathematical point of view, the limit value of the QSA matrix for very small stimulations is consistent with local analysis and represents a quadratic transfer function as a Volterra kernel. For larger stimulations, the quadratic response becomes dependent on the stimulation and the ratio (Equation 1) cannot be interpreted as a transfer function. However, the nonlinear output is still a quadratic signal without higher order frequency contamination (Magnani and Moore, 2011). In fact, larger low peak stimulations evoke the low frequency range near the resonance, in which interactions |*f_i_* − *f_j_*| corresponding to the enhanced corners are generated. Such an interpretation is supported by the enhanced output amplitudes of the interactive difference frequencies in Figure 2C (inverted triangles). This may increase the quadratic response at these interactions, relative to the stimulation.

These nonlinear effects are present at the usual membrane potential excursions in the mV range and appear amplified for stimulations tuned to the preferred frequency response of a particular neuron. Interestingly, other constant stimulation profiles (not represented) lead to quadratic functions independent of stimulation amplitudes for membrane potential responses up to a few mV. For stimulation profiles with enhanced low frequencies, this independence of stimulation amplitude occurs only for a tenfold lower response amplitude (10–100 microvolts), which again emphasizes the sensitivity of the nonlinear responses to the frequency content of the stimulation.

These results are consistent with experimental data of Figure 6 showing that low frequency stimulation leads to an enhancement of high frequency interactions for both QSA matrix and *R* function. Figure 8 indicates that high frequency interactions elicited by low frequency stimulation can nearly disappear if the stimulation peak is shifted to higher intermediate frequencies. In both cases, the linear responses remains identical despite major differences in the nonlinear behavior.

### 3.5. Dendritic stimulation

**Figure 10:**
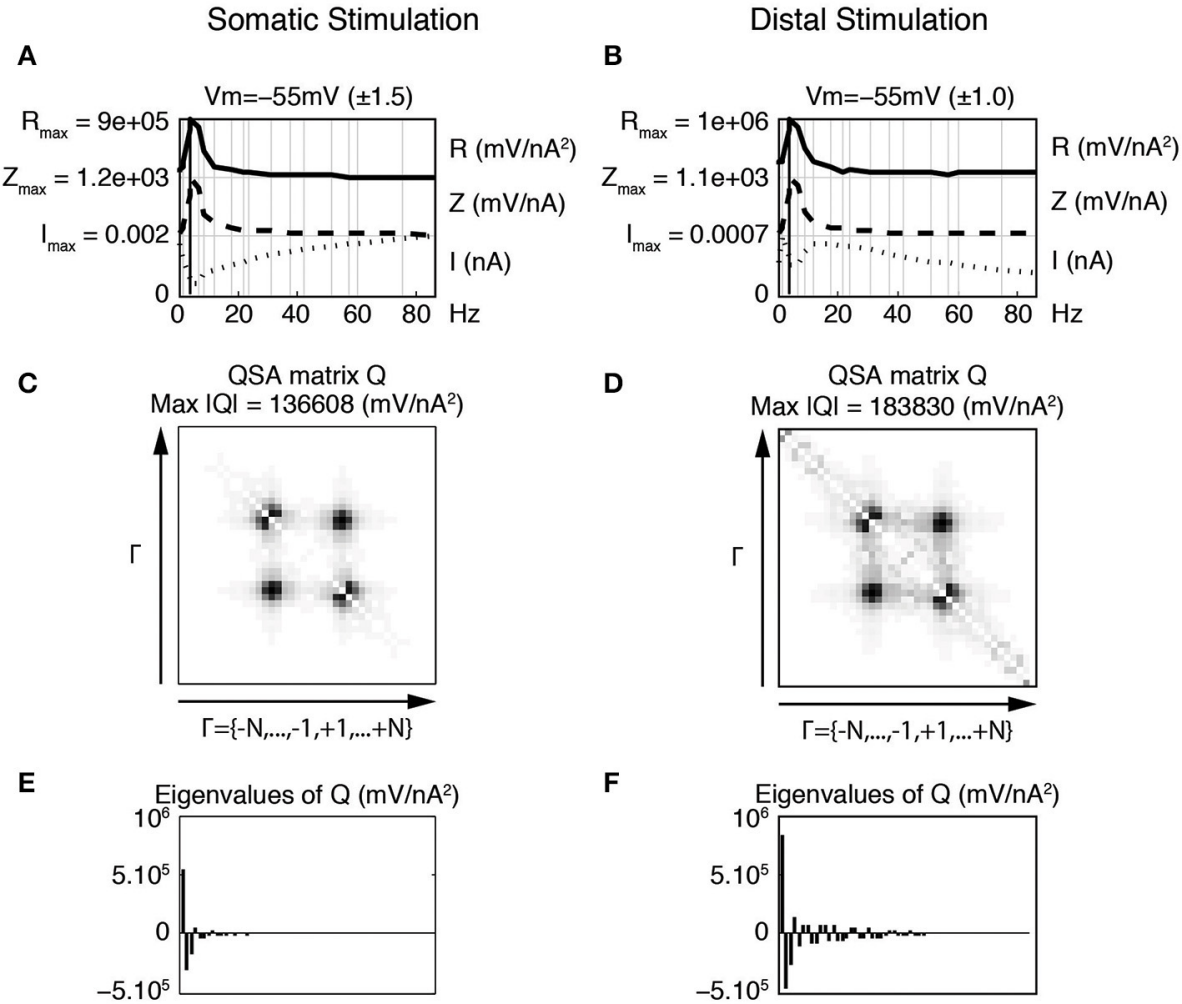
**Effect of distal vs. proximal flat stimulation on somatic nonlinearities**. The linear and quadratic functions were calculated using only the current passing across the soma membrane, as opposed to the total injected current. Thus, the analyses apply to the soma independent of the location of the stimulating electrode. Left column **(A,C,E)** Responses to the flat stimulation of the somatic compartment. Right column **(B,D,F)** Responses to the flat stimulation applied at the most distal compartment. **(A,B)** Juxtaposed plots with respect to the input frequencies, namely the amplitudes of current stimulation *I*, linear impedance *Z*, and *R* function. The resonance of the impedance is *f*_6_ = 4.0 Hz for both stimulations. **(C,D)** Amplitudes of the coefficients of the QSA matrix *Q* with the maximum value given by Max |*Q*|. **(E,F)** Eigenvalues of the QSA matrix. All parameters of the model neuron are the same as in Figure 7.

Since cable properties of dendrites induce a signal filtering, the marked effect of the stimulation content on the nonlinear response could play a role in how dendrites process signal inputs. It can be expected that distal vs. proximal synaptic inputs to single neurons would lead to different responses in the soma. Figure 10 illustrates such effects with model simulations. It compares the soma potential response to a current stimulation in the soma (left column) vs. a stimulation of the most distal dendritic compartment (right column). In both cases, the stimulation profile is flat but the represented current *I* is not the stimulation but the computed current flowing across the soma membrane.

In order to compare the two approaches, a somatic impedance was calculated by determining the ratio of the soma potential and the current flowing across the soma membrane, rather than the total injected current typically used for either point or transfer impedances. This procedure determines just the impedance of the somatic compartment in isolation, which is nearly independent of the stimulation profile, as shown in Figure 10. At smaller stimulations, the two impedances converge to identical functions. Thus, this approach can be used to analyze the effect of dendritic filtering of the injected current from the dendrite on the nonlinear responses of just the soma.

In general, the computed impedance for the somatic compartment is higher than the impedance of the soma plus the dendrite. This occurs because the impedance of a single somatic compartment is greater than the soma with attached dendritic compartments (not shown). More precisely, a significant portion of the injected current passes down the dendrite leaving a smaller current for the soma, which has a minimum value near the resonance frequency, at which the impedance is by definition maximal. Thus the smaller somatic current with the same soma potential gives a greater impedance. The total injected current would be the sum of the somatic current and remaining current flowing down the dendrite.

When a flat stimulation is injected at the end dendritic compartment (Figure 10B), the somatic current has a minimum near the resonance frequency and the somatic impedance is nearly the same to that determined with somatic stimulation. The simulations show that the nonlinear responses (QSA matrix, *R* function) are greater for the distal stimulation (right column) due to the dendritic filtering, which can be seen to be a Gaussian-like peak around 15 Hz.

When stimulating the end dendritic compartment, the other compartments significantly reduce the high frequency content of the current reaching the soma (Figure 10, right column). This current increases from a minimum at the resonant frequency to a maximal value and then progressively decreases with frequency as would be expected from dendritic filtering. These effects are the consequence of the progressive filtering of the membrane potential by each dendritic compartment toward the soma. Each individual dendritic compartment shows similar scaled linear behavior, which is dependent on the potential in each compartment as determined by the cable structure including the voltage dependent channels.

The QSA matrix for the dendritic stimulation shows enhanced corners that are similar to those observed for filtered stimulations applied to the soma in Figures 6, 8. However, the dominating responses at the intermediate harmonics and interactive difference frequencies are comparable for both somatic and dendritic stimulations. Decreasing the stimulation amplitudes leads to the same limit functions for impedances and QSA matrices (not shown) as found for the Gaussian-like stimulations of Figure 9.

Figure 10D also shows peaks for the QSA matrix at harmonics and high frequency interactions (around 2 *f*_6_ = 8.0 Hz and *f*_20_ − *f*_19_ = 11 Hz). These are similar to those observed for the Gaussian-like window of Figure 8, which has peaks clustered around harmonic frequencies of *f*_7_ = 6.4 Hz and *f*_8_ = 8.7 Hz.

Thus, the dendritic filtering is simulated by a Gaussian-like stimulation applied to the soma, which is related to the effect of a filtered stimulation applied to the soma as in Figures 6, 7. In summary, these results support the fact that the enhancement of low frequency amplitudes increases nonlinear responses.

Increasing the electronic length leads to somatic current profiles similar to the filtered stimulations of Figures 6, 7 and QSA matrices that are quite similar. From a physiological point of view, these nonlinear effects due to filtering are very much dependent on the precise dendritic location of the stimulation and appear to be present at all input amplitudes. However, these nonlinearities are not significant until the soma potential responses are in the mV range where they also contribute to a distortion of the linear behavior if the frequency content of the stimulation is overlapping. Clearly, higher order nonlinearities are also generated with very large stimulations.

### 3.6. Spike frequency modulation

The subthreshold nonlinear responses are markedly dependent of the membrane potential and essentially reach their maximum values just below the threshold. This would suggest that the nonlinear behavior at just below the threshold membrane potential could provide a reasonable estimation of the suprathreshold action potential response. In order to directly determine suprathreshold responses, a QSA analysis was done on action potential responses using three or four stimulation frequencies near the resonance, namely 0.3, 1.3, and 3.5, or 4, 6.4, 8.7, and 12.1 Hz, the latter encompassing the resonance range of frequencies. Both linear and quadratic analyses were done using a Fourier analysis of unit spike events constructed from their peak values, which extracts the expected low frequency responses.

**Figure 11:**
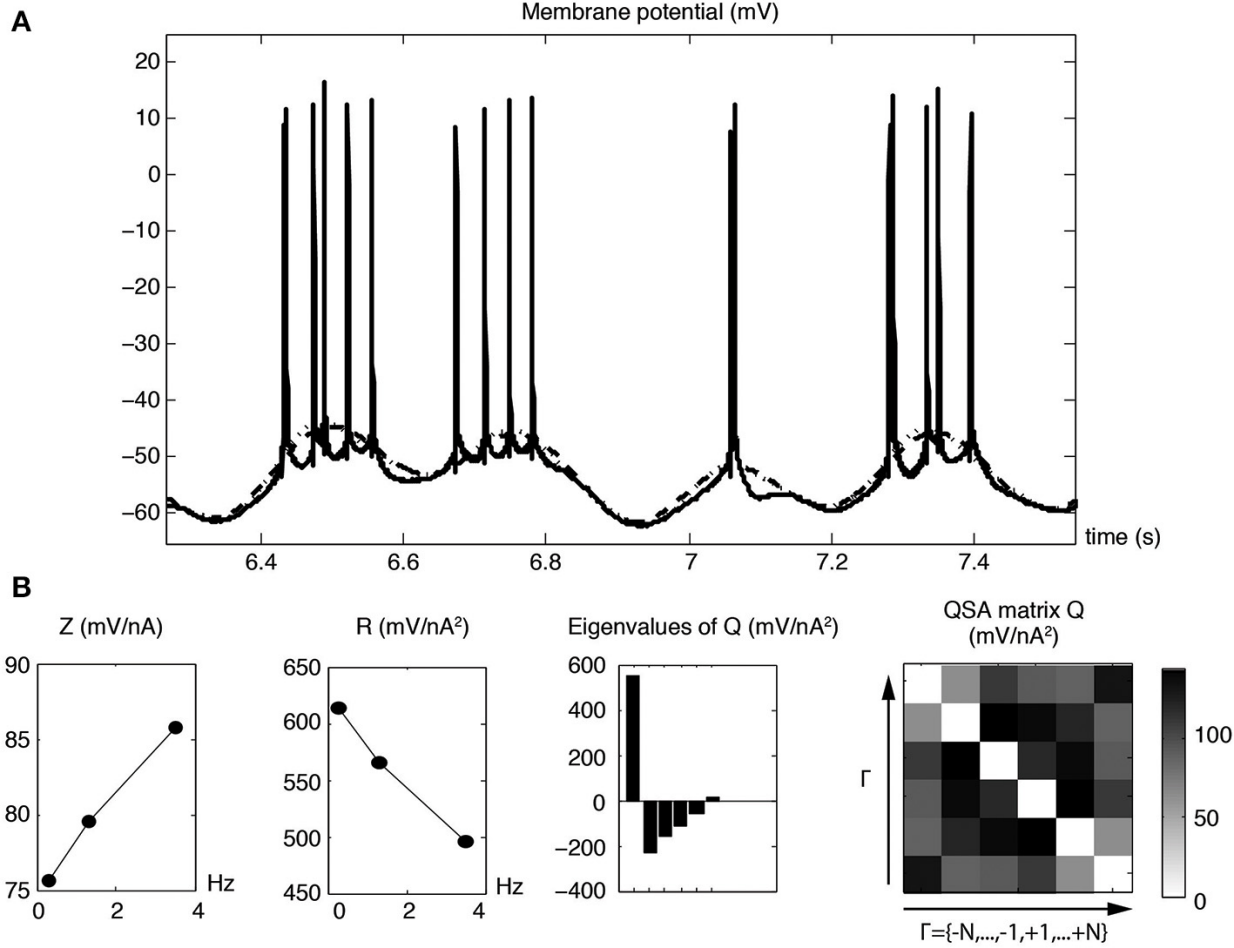
**Linear and quadratic membrane potential responses for pre-resonance stimulation frequencies (*f*_1_ = 0.3, *f*_2_ = 1.3 and *f*_3_ = 3.5 Hz). (A)** Membrane potential responses (solid line) with modulated action potential responses. The dashed and dotted curves show the linear and quadratic responses of the underlying membrane potential for the three stimulating frequencies. **(B)** Linear and quadratic analyses for a subthreshold response displaced by 0.5 mV without action potentials. The corresponding reconstructed time domain analyses are almost identical to those shown in the first row.

Figure 11A shows modulated action potential responses to a multi-sinsuoidal stimulation containing three frequencies (0.3, 1.3, and 3.5 Hz) that are all below the resonance. The underlying traces show the linear and quadratic membrane potential reconstructions for just these frequencies, which are sufficiently low to minimize the contamination of the Fourier transform by the shape of action potentials. Figure 11B represents linear and quadratic analyses of the membrane potential for the same neuron stimulated just below threshold. Since there are few frequencies, the superimposed reconstructed linear and quadratic responses have slight differences (Figure 11A, dashed linear vs. dotted quadratic lines). However, the quadratic responses remain significant (Magnani and Moore, 2011) to encode fundamental nonlinear properties of the neuron that cannot be predicted by linear analysis. The linear impedance shows three rising values that precede the usual resonance of stellate neurons. The QSA matrix and the *R* function indicate that both low and higher frequencies near the harmonics have the greater interactive responses, which are also chararactized by a dominant eigenvalue.

**Figure 12:**
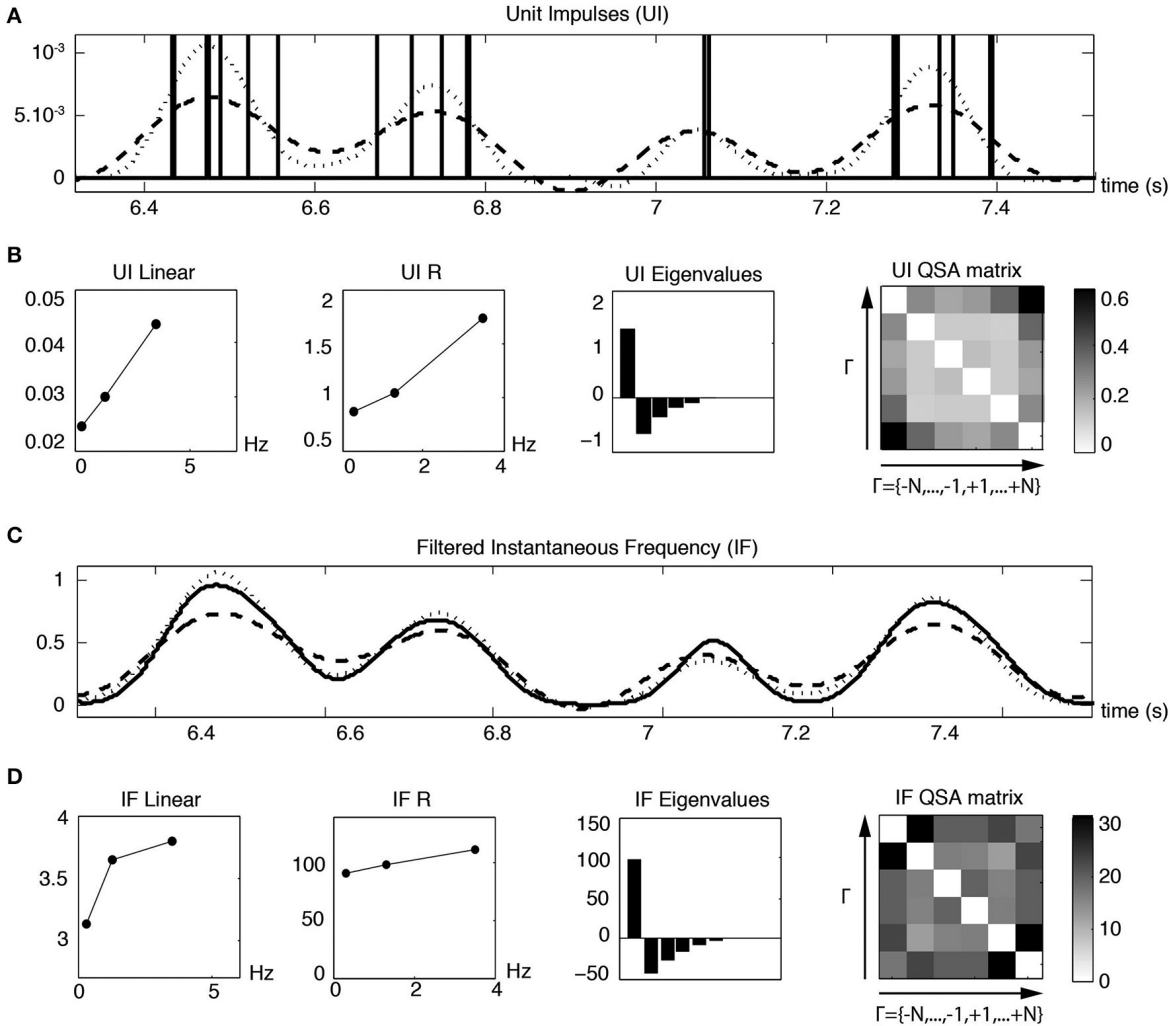
**Linear and quadratic responses of unit impulses (UI) to pre-resonance stimulation frequencies (*f*_1_ = 0.3, *f*_2_ = 1.3 and *f*_3_ = 3.5 Hz). (A)** Unit impulses corresponding to each action potential event of Figure 11. The dashed and dotted lines are the reconstructed linear and quadratic components based on the analysis of the second row. **(B)** Linear and quadratic analyses computed from the Fourier transform of the unit impulses. **(C)** The solid line is a slightly Gaussian filtered instantaneous frequency (IF) trace constructed from the spike times. The dashed and solid lines are the reconstructed linear and quadratic components based on the analysis of the fourth row. The Gaussian filter is sufficient to remove peaks related to spike times. The analysis of less filtered IF curves (not shown), such that distorted spike events are visible, is very similar to scaled unit impulses analyses. **(D)** Linear and quadratic analyses computed from the Fourier transform of the filtered instantaneous frequency.

The time instants of each action potential of Figure 11A were converted to unit impulses (UI), as illustrated in Figure 12A. The dashed and dotted traces show the linear and quadratic analyses computed from the Fourier transform of the unit impulses. Figure 12A shows the waveforms of both the linear and quadratic analyses of the unit impulses, which are similar to those of the membrane potential of Figure 11A. The QSA matrix and the *R* functions are slightly different, showing a greater amplitude for the highest frequency interactions, mainly the highest harmonic frequencies 2 *f*_3_ = 7 Hz. It is evident from Figure 12A that, when the frequency of spiking modulation is high, namely the dotted quadratic response has a much greater amplitude than the dashed linear line. Also, both the membrane potential and unit impulses analyses have a dominant eigenvalue, which suggests that they have features in common for their nonlinear behavior.

Since the Fourier analysis of a spike train contains many high frequency components unrelated to the lower modulated spiking frequencies, it is useful to construct an instantaneous frequency (IF) curve from the spike times. A slightly Gaussian filtered IF curve is shown in Figure 12C as a solid trace for the same time range illustrated for the unit impulses in Figure 12A. The dashed linear and dotted quadratic traces are the reconstructed responses of the solid trace. The linear and quadratic analyses are shown in Figure 12D and are similar to the analysis of the unit impulses in Figure 12B except for the details of the QSA matrix. The QSA matrix of unit impulses shows high frequency interactions at sums *f_i_* + *f_j_* and harmonics 2 *f_i_*. The QSA matrix of instantaneous frequency curve has increased amplitudes of high frequency interactions at differences |*f_i_* − *f_j_*|. These various effects are also reflected in the time domain of the reconstructed responses (first row compared to third).

**Figure 13:**
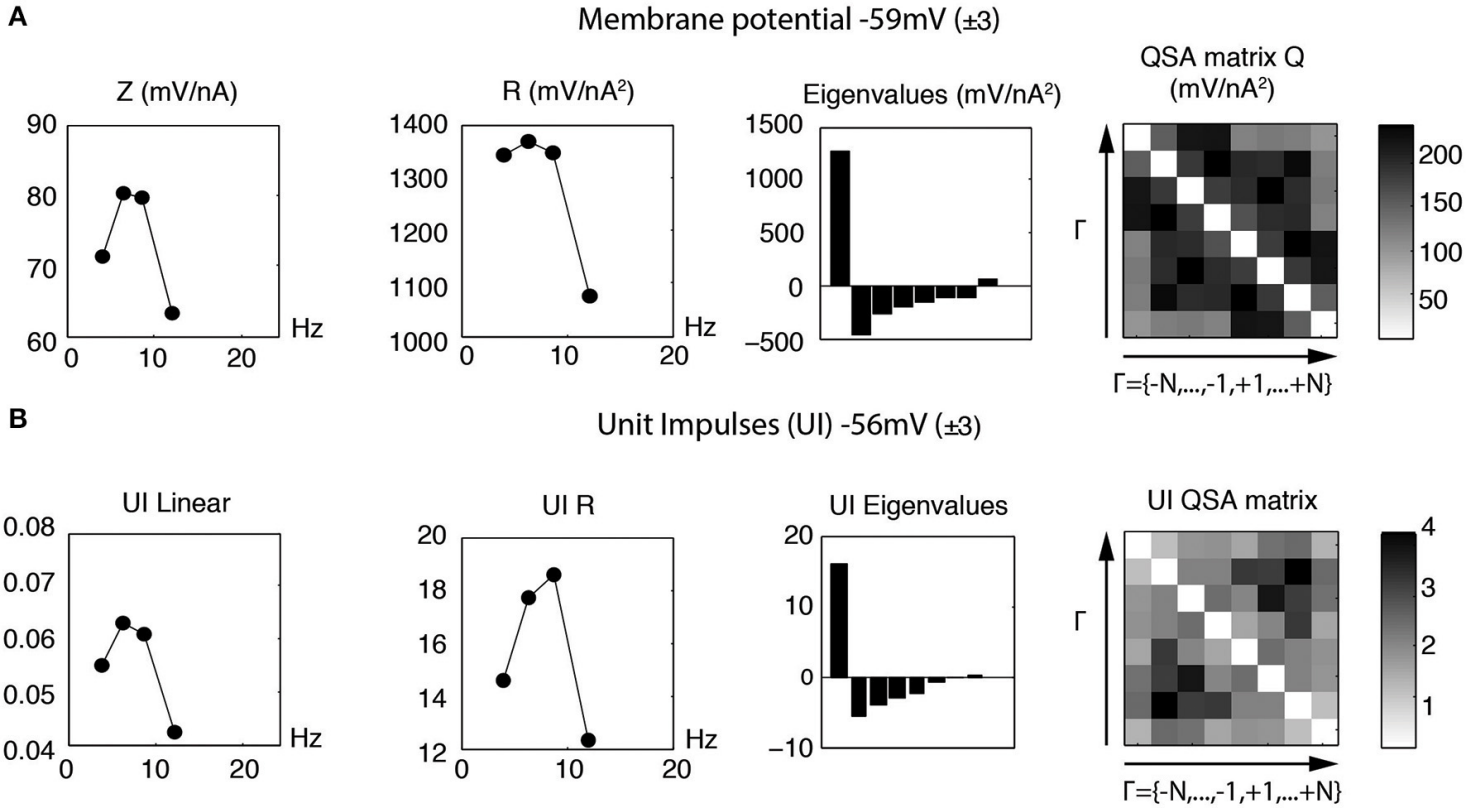
**Linear and quadratic analyses of membrane potential and unit impulses for stimulation frequencies near the resonance (*f*_1_ = 4, *f*_2_ = 6.4, *f*_3_ = 8.7 and *f*_4_ = 12.1 Hz). (A)** Analysis of subthreshold membrane potential. **(B)** Analysis of suprathreshold unit impulses. The linear and quadratic behaviors for the subthreshold and suprathreshold responses have similar form for these resonance frequencies.

Figure 13 illustrates linear and quadratic analyses of the membrane potential (Figure 13A) measured without action potentials compared to unit impulses (Figure 13B), for a stimulation based on four frequencies (*f*_1_ = 4, *f*_2_ = 6.4, *f*_3_ = 8.7 and *f*_4_ = 12.1 Hz) that encompass the resonance of a stellate neuron. It is apparent that both the linear and nonlinear behaviors are quite comparable in this frequency range. The quadratic responses associated with the middle two frequencies are enhanced for both the membrane potential and unit impulses analyses. In general, the QSA matrix of unit impulses shows a more restricted set of intermediate frequency interactions than observed for the QSA matrix of the membrane potential, as can be observed on Figure 13. Nevertheless, the similarity of the linear and quadratic analyses of the subthreshold membrane potential and unit impulses are striking. This suggests that the suprathreshold modulated spiking behavior is reasonably well approximated by the subthreshold membrane potential nonlinearity, just below threshold. As threshold is approached, the subthreshold responses take on a more nonlinear character, which finally determines the actual unit impulses behavior, albeit in combination with the linear behavior.

## 4. Discussion

The firing properties of the MEC stellate cells play a critical role in their function as part of the grid cell network. These neurons show oscillatory and nonlinear properties that are likely to be involved in the operation of networks involved in spatial awareness. We have used a novel theory, quadratic sinsoidal analysis (QSA), to rigorously determine the nonlinearity of SCs (stellate cells) near threshold for a direct comparison with linear behavior. This multi-sinusoidal frequency probing not only provides a quantitative measurement of these properties through the QSA matrix, but also an algebraic characterization of the quadratic function as a Hermitian operator. The nature of the quadratic responses are significantly different than subthreshold linear behavior and gives an indication of the distinctive differences between sub- and suprathreshold responses in SC neurons (Haas et al., 2007). We have shown that at physiological levels of stimulation, neurons and their models generate significant responses at harmonic and interactive frequencies that are not present in the input signal. Thus, the quadratic responses contain more frequencies over a wider frequency band than the input signal. As a consequence, they provide significant amplification at dynamically changing membrane potentials.

Previous studies have shown that the nonlinear responses measured in neurons appears to be dominated by the dendrites (Magnani and Moore, 2011), which receive the bulk of the synaptic input. It is likely that the principal source of nonlinearity in stellate neurons near threshold is the persistent stochastic sodium conductance (*g*_NaP_), which has been shown to be the primary source of channel noise in these neurons (Dorval and White, 2005). Thus, dendritic processing plays an important role in the firing behavior of stellate neurons in a number of ways (Zhuchkova et al., 2013). For example, the inherent filter properties of cable structures in combination with their active voltage dependent channels alter the profile of the stimulation reaching the soma. We have shown that dendritic filtering greatly alters the nonlinear membrane potential response at the soma, which will depend on both the spatial location and frequency content of the stimulation. Finally, we have demonstrated that quadratic analysis of modulated spiking behavior has significant similarities to membrane potential nonlinearities when the membrane potential is just below threshold.

In conclusion, this analysis indicates that subthreshold linear and nonlinear responses are similar to suprathreshold firing behavior. Thus, the combined linear and nonlinear behaviors near threshold of the membrane potential are reasonable estimates of suprathreshold behavior given by spike frequency modulation. Linear and nonlinear behaviors a few millivolts below the threshold membrane potential are quite different with the nonlinear component being significantly reduced. The linear components of stellate neurons, such as resonance, are present at membrane potentials hyperpolarized to threshold despite the diminishing nonlinearities. In these membrane potential ranges, the linear resonance behavior is essentially due to the H current (Giocomo and Hasselmo, 2009). There is also a nonlinear component of the H conductance that can be measured at hyperpolarized values, however it is much smaller than nonlinear effects of the sodium conductances near threshold (Magnani et al., 2013). An additional effect of H conductances could occur if they are present in the dendritic tree. In this case, distal inputs could show bandpass resonance characteristics that would propagate to the soma involving both active and passive dendritic filtering, and further increase a nonlinear soma response.

## Author contributions

Christophe Magnani and Lee E. Moore developed the QSA theory and implemented the analysis in MATLAB. Christophe Magnani, Lee E. Moore, John A. White, and Michael N. Economo designed the experiments. Michael N. Economo did the whole cell patch clamp experiments. Lee E. Moore, Christophe Magnani, John A. White, and Michael N. Economo interpreted the results of the experiments. Lee E. Moore and Christophe Magnani did the model simulations, analyzed data and drafted the manuscript. Lee E. Moore, Christophe Magnani, John A. White, and Michael N. Economo edited and revised manuscript.

### Conflict of interest statement

The authors declare that the research was conducted in the absence of any commercial or financial relationships that could be construed as a potential conflict of interest. The reviewer Michael E. Hasselmo declares that, despite having collaborated with co-author John A. White, the review process was handled objectively.
